# Remote Plasma Selective Silicon Etching Enabled Tunable Sub-Fin Process for Improved Parasitic Bottom Channel Control in Gate-All-Around Nanosheet Field-Effect Transistors

**DOI:** 10.3390/nano16010013

**Published:** 2025-12-21

**Authors:** Jiayang Li, Yuan Gao, David Wei Zhang

**Affiliations:** 1 College of Integrated Circuits, Micro-Nano Electronics, Fudan University, Shanghai 200433, China; jiayangli21@m.fudan.edu.cn; 2 State Key Laboratory of Materials for Integrated Circuits, Shanghai Institute of Microsystem and Information Technology, Chinese Academy of Sciences, 865 Changning Road, Shanghai 200050, China; gaoy@mail.sim.ac.cn; 3 School of Microelectronics, Fudan University, Shanghai 200433, China

**Keywords:** remote plasma etching, nanosheet FET, Sub-Fin parasite channel, leakage, thermal conductance, buried oxide, TIS

## Abstract

The parasitic Sub-Fin, beneath the stacked nanosheet FETs, limits both leakage and heat dissipation, acting as the bottleneck for improving the performance of NS-FETs. A Sub-Fin edit technology based on remote plasma etching is proposed to modulate the formation of the Sub-Fin. By controlling the process parameters, the Sub-Fin profile can be continuously modulated from “arrow-shaped” to “bell-shaped,” which provides the flexibility to improve the thermal resistance and reduce the parasitic Sub-Fin-induced degradation, making it suitable for low-power and high-performance applications, respectively. The Sub-Fin edit technology is fully compatible with mature Gate-All-Around (GAA) fabrication processes and offers a feasible approach to balancing the trade-off between Sub-Fin degradation and heat dissipation through the Sub-Fin.

## 1. Introduction

Gate-All-Around (GAA) Nanosheet FETs (NS-FETs) are widely recognized as the mainstream architecture for logic devices in the post-FinFET era [1,2,3,4,5]. Compared to traditional FinFETs, NS-FETs offer superior electrostatic control, effectively suppressing the short-channel effect (SCE) and drain-induced barrier lowering effect (DIBL) [1,6,7]. Their multi-layer stacked nanosheet structure provides higher drive current per footprint while maintaining excellent subthreshold characteristics and scalability, and demonstrates high compatibility with existing high-k/metal gate (HKMG) and Si/SiGe epitaxial processes [8]. However, in GAA structures, the bulk-Si beneath the bottom-most nanosheet is prone to forming a parasitic bottom channel or parasitic bottom transistor (trPBT), leading to issues such as enhanced gate-induced drain leakage (GIDL), threshold voltage ($V_{T}$) shift, and increased off-state current ($I_{off}$), thereby compromising the electrical stability and reliability of the device [9,10,11]. To suppress these parasitic effects, various structural and process solutions have been proposed, including highly doped Punch-Through Stopper (PTS) layers, full bottom dielectric isolation (Full BDI), and partial bottom dielectric isolation (Partial BDI) structures.

The highly doped PTS layer effectively suppresses bottom parasitic conduction, induces higher band-to-band tunneling (BTBT), and GIDL effect, degrades carrier mobility, and causes fluctuations in electrical device uniformity [12,13]. The Full BDI introduces a continuous dielectric layer between the channel and substrate, completely blocks the bottom parasitic path and somewhat reduces GIDL [14]. However, the presence of the dielectric layer impairs the transistor’s heat dissipation capability, resulting in increased lattice temperature and mobility degradation [15,16,17]. The Partial BDI method attempts to balance parasitic suppression with thermal management. Although it has shown some effectiveness in both simulation and experiment [18,19,20], its implementation typically requires complex pattern transfer and multiple etching steps, presenting challenges in process compatibility, complexity, and manufacturability, making it difficult to integrate into advanced circuit fabrication process [15,18,19,20].

To further improve bottom parasitic control and thermal performance, Pohang University of Science and Technology (POSTECH) proposed the Trench Inner-Spacer (TIS) structure in 2023 [15]. This approach forms a dielectric layer on the trench sidewall to prevent source/drain dopant diffusion into the PTS region, thereby compromising the bottom parasitic conduction path. Simulation results show that the TIS structure offers great process tolerance under source/drain over-etch conditions and leading to lower lattice temperature and thermal resistance compared to the BOX isolation method. However, the electrostatic shielding effect of TIS is primarily concentrated on the sidewall region (affecting the transistor access region), while the bottom channel path is not completely blocked. Consequently, devices still suffer from $V_{T}$ instability and residual GIDL. Furthermore, this method requires complex processes performed before epitaxy, including multiple high-precision etchings, selective SiGe etching, and dielectric recesses, demanding extremely high alignment accuracy, which increases manufacturing complexity and limits process feasibility.

In contrast, the Institute of Microelectronics of the Chinese Academy of Sciences proposed the Narrow Sub-Fin technology, which enhances bottom electrostatic control by reducing the Sub-Fin width, effectively suppressing parasitic bottom conduction. 3D TCAD simulation results indicate that this structure can reduce GIDL by approximately 70%, improve the $I_{\mathrm{on}}$/$I_{\mathrm{off}}$ ratio by over 20%, and achieve a good balance between leakage and thermal resistance [19]. However, this method remains at the simulation stage. The proposed process paths based on Reactive Ion Etching (RIE) or Atomic Layer Etching (ALE), which lack sufficient etch selectivity in Si/SiGe multilayer stacks, potentially causing damage to the SiGe layers and subsequently affecting device reliability and gate control stability.

In summary, existing structural and process optimization methods for suppressing the bottom parasitic channel in GAA NS-FETs struggle to simultaneously meet three key requirements: (1) effectively inhibiting parasitic bottom transistor (trPBT) leakage; (2) maintaining great heat dissipation; and (3) possessing compatibility and manufacturability with advanced Si/SiGe epitaxial processes.

Specifically targeting the aforementioned challenges, this work proposes and experimentally validates a tunable Sub-Fin structure process, called Sub-Fin edit technology, based on Remote Plasma Etching (RPE). By optimizing the ${\mathrm{CF}}_{4}/O_{2}/N_{2}$ gas ratio and etch time, it enables highly selective isotropic etching of Si, allowing precise adjustment of the formation of the Sub-Fin from “arrow-shape” to “bell-shape” and its Bottom Deposition Layer (BDL) with virtually no damage to the SiGe layers. Therefore, various geometric structures for different applications could be realized by the Sub-Fin editing technology. This structurally mitigates the bottom parasitic conduction path while preserving a continuous silicon thermal conduction path, improving the thermal resistance, and reducing the parasitic Sub-Fin-induced degradation. Compared to existing TIS and BDI methods, this method avoids multiple high-precision etching and complex alignment steps while maintaining high Si/SiGe selectivity and low damage characteristics, exhibiting superior process compatibility and manufacturing feasibility. This research provides a manufacturable and tunable Sub-Fin/BDL process path for solving the bottom parasitic conduction problem in GAA Nanosheet FETs. It marks the first successful translation of the simulated “arrow-shaped” Sub-Fin structure into a practically achievable process module, laying the foundation for the future design of high-performance, low-leakage, and high-thermal-reliability devices.

## 2. Materials and Methods

In this study, a commercially available multilayer stacked Si/SiGe superlattice wafer was used to characterize the Si selective etching process. The wafer consists of Si layers with a thickness of 30 nm, SiGe layers with a thickness of 50 nm, and a Ge content of 25% in the SiGe layers. The Si/SiGe superlattices were epitaxially grown on Si substrates using Reduced Pressure Chemical Vapor Deposition (RPCVD) equipment, ensuring that the SiGe layers share the same crystal structure and closely matched lattice constants as the Si substrate. As a result, the Si layer is subjected to tensile stress, while the SiGe layer is subjected to compressive strain. The Si selective etching process was carried out on a 200 mm etching platform consisting of two reactors connected via a vacuum transfer chamber: an inductively coupled plasma (ICP) reactor for anisotropic etching of the hard mask and Si/SiGe superlattices, and a Remote Plasma Source (RPS) Reactor for isotropic and selective etching of the Si. In the RPS reactor, the plasma is generated by the application of a microwave discharge (2.45 GHz) in a quartz tube. A showerhead separates the substrate from the plasma, blocking charged species while allowing neutral particles to pass through the holes in the shower plate. Therefore, the substrate is exposed solely to reactive neutrals, allowing isotropic and chemical-type etching.

**Figure 1:**
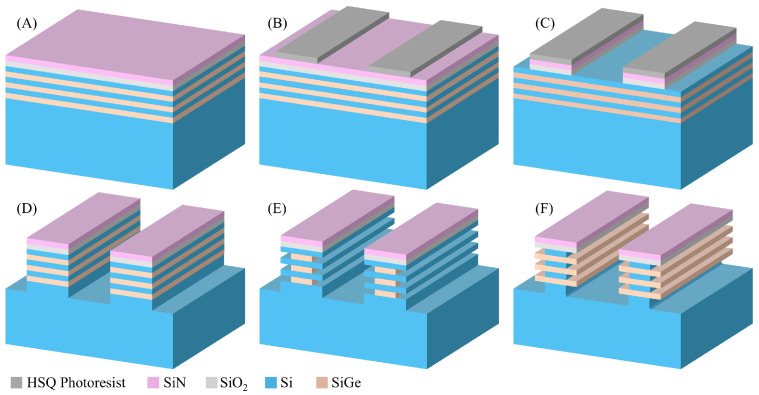
Process flow and test structure for evaluating Si-selective etching in Si/SiGe superlattices. (**A**) Si/SiGe superlattice and ${\mathrm{SiO}}_{2}$/SiN hard mask, (**B**) fin lithography, (**C**) ${\mathrm{SiO}}_{2}$/SiN hard mask etch, (**D**) fin etch, (**E**) Selective etch for SiGe, (**F**) Selective etch for Si.

The test structure and process flow that were designed for the study of the Si selective etching process are shown in Figure 1. The pattern structures were fabricated using the following process: first, a 60 nm-thick SiN layer and a 90 nm-thick ${\mathrm{SiO}}_{2}$ layer were sequentially deposited on a Si/SiGe superlattice wafer using plasma enhanced chemical vapor deposition (PECVD) to form the hard mask (Figure 1A). Electron beam lithography (EBL) was then used to define the patterns, which were designed as rectangular arrays with a pitch of 1 μm, a length of 20 μm, and a width of 500 nm (Figure 1B). After that, the patterns were transferred sequentially to the hard mask and Si/SiGe superlattices using anisotropic etching processes, allowing the buried Si and SiGe layers to be exposed to the etching species (Figure 1C,D). The etching of the ${\mathrm{SiO}}_{2}$ hard mask was performed using plasma generated from a ${\mathrm{CF}}_{4}/{\mathrm{CHF}}_{3}/O_{2}/\mathrm{He}$ gas mixture, while the SiN hard mask was etched with plasma from a ${\mathrm{CHF}}_{3}/O_{2}/\mathrm{He}$ gas mixture. The etching of the Si/SiGe superlattices involved ${\mathrm{CF}}_{4}$ for the breakthrough step, followed by ${\mathrm{Cl}}_{2}/\mathrm{HBr}/\mathrm{He}$ for the main etch. The anisotropic etching processes were performed in the ICP reactor. To remove remaining by-products and the oxidized layer on the pattern surface, $O_{2}$ plasma-based dry etching and wet cleaning (dip in 1% DHF for 60 s) were performed. Finally, the Si/SiGe superlattices, cut into 2×2 ${\mathrm{cm}}^{2}$ coupon wafers, were fixed on an 8-inch carrier wafer and transferred to the RPS reactor for SiGe selective etching (Figure 1E) and Si selective etching (Figure 1F). In the RPS reactor, the wafer is placed on an electrostatic chuck, which regulates the requisite temperature for the etching. It is notable that a number of process parameters were maintained across all studies. For instance, the microwave source power was set to 1000 W, the chamber pressure was regulated to 1.2 Torr, and the chuck temperature was maintained at 25 °C.

Leveraging the multilayer Si/SiGe stack as a test structure, it is possible to observe and evaluate the selectivity and etching rate of the process by scanning electron microscopy (SEM) and transmission electron microscopy (TEM). The etch amounts were determined by measuring the difference in the remaining Si and SiGe before and after the Si selective etching process, which was then used to calculate the etching rate and selectivity.

## 3. Results

### 3.1. A Novel Method for Si Selective Etching

**Figure 2:**
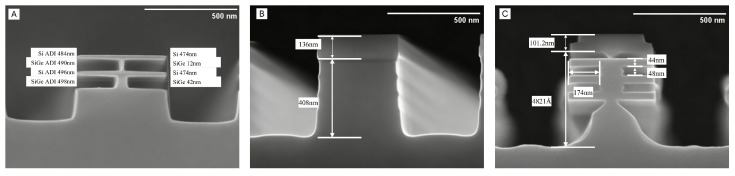
Etch results with tunable Si/SiGe selectivity Sub-Fin edit technology. (**A**) Selective etch for SiGe with unchanged Sub-Fin, (**B**) Low-selectivity etching for Fin, (**C**) Selective etch for Si with “Bell shape” Sub-Fin.

In mainstream GAA fabrication processes, the parasitic Sub-Fin beneath the GAA FET has attracted widespread attention. However, conventional selective etching methods, which can only remove SiGe from the Si/SiGe superlattice, face the challenge of efficiently etching Si and modulating the Sub-Fin. The structure of the etched superlattice based on conventional selective etching methods is shown in Figure 2A.

In our previous study, a highly selective Si/SiGe etching process was established in a Remote Plasma Etching (RPE) system. The structure of the etched superlattice based on novel selective etching methods is shown in Figure 2B,C. By introducing $N_{2}$ into the ${\mathrm{CF}}_{4}/O_{2}$ plasma, the generated NO radicals selectively disrupt the oxide passivation layer on the Si surface and accelerate the chemical etching of Si. In contrast, the SiGe layer, protected by a stable oxide layer, shows a significantly lower etch rate, thus yielding a high Si/SiGe etch selectivity [21,22]. This result is attributed to the NO radical-dominated selective etching mechanism and demonstrates the high selectivity and low-damage characteristics of the RPE process in Si/SiGe multilayer structures [23].

Based on the established highly selective Si/SiGe etching process, the ${\mathrm{CF}}_{4}/O_{2}/N_{2}$ gas ratio is optimized to achieve controllable isotropic etching of the Sub-Fin while maintaining sufficient Si/SiGe selectivity. The Si/SiGe selectivity etch rate is calculated by:

(1) $$r=\frac{2A}{C-B}$$

where *A* represents the etch depth of the Si layer, *B* represents the thickness of the remaining SiGe structure after etching, and *C* represents the total thickness before etching. By reducing the $O_{2}$ flow rate to 200 sccm for increasing the F:O ratio in the etching environment, the Si etch rate increased to 4.2 nm/s, while the Si/SiGe selectivity slightly decreased to about 46 (Figure 2C). The reduction in $O_{2}$ content suppressed the oxidation/deposition reactions, mitigating excessive passivation on the Si surface and allowing $F^{*}$ radicals to participate more effectively in the etching process, thereby accelerating Si removal. This strategy successfully yields a “Narrow Sub-Fin” structure, providing a process window for fine-tuning the bottom structure.

**Figure 3:**
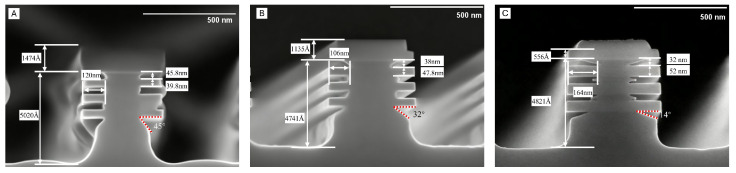
High selectivity Si etch induced etch angle. (**A**) sample with 45° angle, (**B**) sample with 32° angle, (**C**) sample with 14° angle.

As shown in Figure 3, the etching results clearly demonstrate a significant reduction in the etch angle between Sub-Fin and the SiGe layer, which is reduced from 45° to around 15° with process optimization, owing to the reduction in $O_{2}$ flow rate. The suppressed $O^{*}$ radical concentration under reduced $O_{2}$ flow limited the oxidation reaction on the Si surface. This modification enabled $F^{*}$ radicals to interact with the Si layer more directly and efficiently, consequently enhancing the etching process. The compressive stress in the Si/SiGe stack, induced by lattice mismatch, creates a stress gradient that governs the spatial etch rate of Si higher near the SiGe interface and lower in the central or top Si layers. Furthermore, the topmost Si layer in the Si/SiGe stack, being near the hard mask location, experiences the least influence from SiGe stress, and accordingly, the lateral etch rate in this region is the slowest. Building on the premise that Si selective etching is initiated at high-stress sites, we further demonstrated that the etch angle can be modulated by adjusting the process parameters. The gas flow rates in the ${\mathrm{CF}}_{4}/O_{2}/N_{2}$ mixture were systematically optimized. Specifically, the $O_{2}$ flow rate was reduced from 400 sccm to 200 sccm, resulting in an increased F:O ratio within the etching chamber.

**Figure 4:**
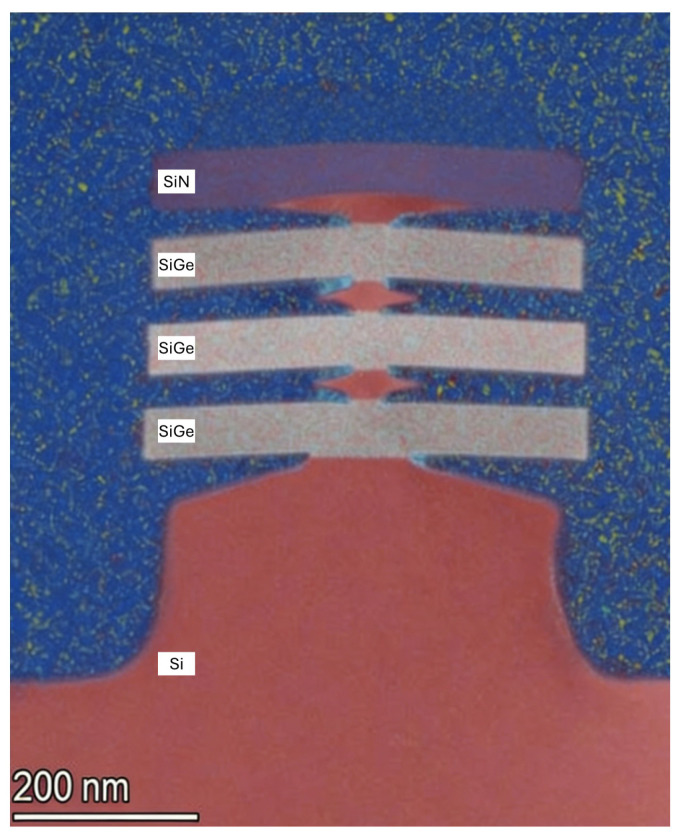
Etching results for the ${\mathrm{CF}}_{4}/O_{2}/N_{2}$ process with a 60-s process time.

Under the optimized conditions of a 25 °C reaction temperature, 1.2 Torr chamber pressure, 1000 W source RF power, gas flow rates of ${\mathrm{CF}}_{4}$/$O_{2}$/$N_{2}$ = 300/400/200 sccm, and a 60 s etch time, the resulting etch profile is shown in Figure 4.

### 3.2. A Novel Method for Sub-Fin Shape Edit

Previous research on Narrow Sub-Fin technology utilized 3D TCAD simulations to investigate the impact of different Sub-Fin narrowing amounts ($T_{N}$) on device electrical characteristics. The simulation results indicate that as $T_{N}$ increases, the parasitic gate-induced drain leakage ($I_{\mathrm{GIDL}}$) is significantly reduced [19]. At $T_{N}=8$ nm, $I_{\mathrm{GIDL}}$ decreases by approximately 70%, and the off-state current ($I_{\mathrm{off}}$) is reduced simultaneously. Meanwhile, the on-state current ($I_{\mathrm{on}}$) remains almost unchanged, leading to an improvement in the $I_{\mathrm{on}}/I_{\mathrm{off}}$ ratio of over 20%. Concurrently, both the subthreshold swing (SS) and gate capacitance ($C_{g}$) are optimized [19]. These results theoretically validate the potential of Sub-Fin engineering in suppressing the bottom parasitic channel and improving electrical characteristics.

**Figure 5:**
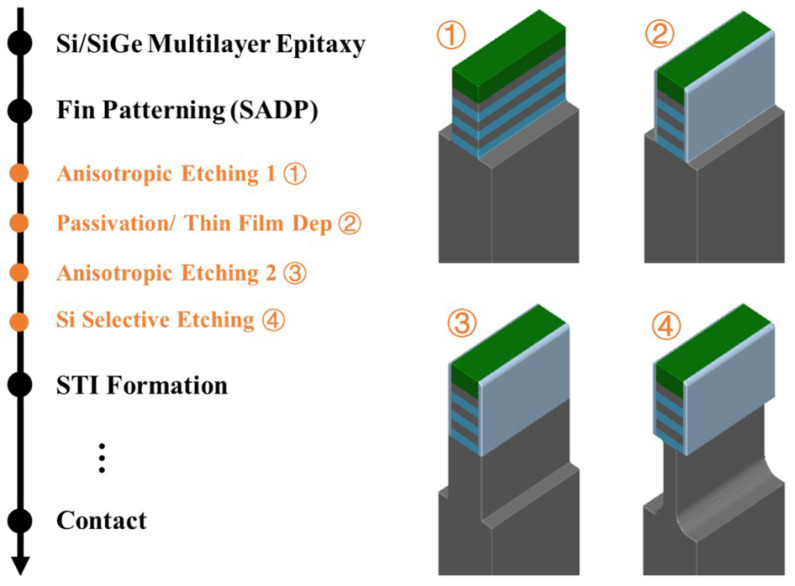
Process flow and 3D schematic of the narrow Sub-Fin bottom dielectric isolation scheme utilizing Si-selective etching.

As shown in Figure 5, the overall process flow of this study remains consistent with the Narrow Sub-Fin structure proposed by the Institute of Microelectronics of the Chinese Academy of Sciences. It introduces additional etching and deposition steps between the Fin Patterning and STI Formation process modules. The detailed process flow is as follows:

1. After achieving the predefined pattern size scaling using multiple patterning technology, an anisotropic etching process is first employed to etch the fins, with the etch depth corresponding to the thickness of the Si/SiGe multilayer stack.
2. Immediately following this, a passivation treatment or a thin-film deposition process is utilized to form a protective layer on the sidewalls of the Si/SiGe multilayer fin structure to ensure the successful execution of subsequent steps.
3. Subsequently, the anisotropic etching process is continued to further deepen the fin etch, forming shallow trenches intended for filling with dielectric material.
4. Finally, and most critically, a Si-selective etching process is employed to precisely and laterally remove the Si material in the Sub-Fin region beneath the Si/SiGe multilayer fin structure. This step effectively narrows the width of the Sub-Fin, where a parasitic bottom channel could potentially form, thereby significantly mitigating its negative impact on device performance.

**Figure 6:**
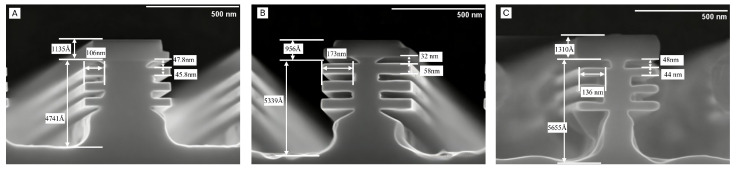
Sub-Fin edit technology fabricated a different shape Sub-Fin. (**A**) broad bell shape, (**B**) narrow bell shape, and (**C**) arrow shape [24].

However, the key difference between the two approaches lies in the etching mechanisms employed. The Narrow Sub-Fin approach proposed by IMECAS, based on RIE or ALE processes, which is an isotropic etch method, offers limited etch selectivity for SiGe material. Consequently, during the Sub-Fin width narrowing process, the underlying SiGe layer is inevitably partially etched or damaged. This leads to the subsequent STI formation, where the dielectric layer intrudes upward along the damaged region, causing morphological distortion in the metal gate control area (as shown in Figure 6C). This distortion triggers electrical instabilities such as threshold voltage shift ($V_{T}$ shift) and enhanced GIDL.

In contrast, this study proposes a Si-selective etching process based on a Remote Plasma Etching (RPE) system, achieving for the first time in experiments a true Narrow Sub-Fin structure. Leveraging the high Si/SiGe selectivity inherent to the RPE system, this method enables lateral shrinkage and morphological reconstruction of the Sub-Fin with virtually no etching of the SiGe layer. By precisely adjusting the etching time and the ${\mathrm{CF}}_{4}/O_{2}/N_{2}$ gas ratio, the lateral width and vertical depth of the Sub-Fin can be flexibly controlled, allowing for precise tuning and engineerable design of the bottom structure.

As shown in Figure 6B, after STI formation, the dielectric boundary is sharp, and the gate region remains intact without any intrusion, while the bottom SiGe layer is perfectly preserved. This fully demonstrates the superiority of this process in terms of structural preservation and etch selectivity. This method not only effectively suppresses the formation of parasitic bottom channels but also preserves a continuous Si thermal conduction path, achieving co-optimization among structural, electrical, and thermal performance.

In the Si selective etching system based on the ${\mathrm{CF}}_{4}/O_{2}/N_{2}$ gas mixture, reducing the $O_{2}$ gas flow rate indirectly enhances the etching effect in the reaction, while increasing the ${\mathrm{CF}}_{4}$ gas flow rate directly enhances the etching effect in the etching reaction. To investigate the effect of ${\mathrm{CF}}_{4}$ gas flow rate on the etching process, multiple ${\mathrm{CF}}_{4}$ gas flow rates were tested. Based on the original process parameters, the ${\mathrm{CF}}_{4}$ gas flow rate was increased from 300 sccm to 400 sccm and 500 sccm, respectively, while the process time was shortened to 30 s. The corresponding etching results are shown in Figure 6. As the ${\mathrm{CF}}_{4}$ gas flow rate increases, when the ${\mathrm{CF}}_{4}$ gas flow rate is 500 sccm, the bottom Si etching becomes significant, while the bottommost SiGe maintains good etching selectivity. Furthermore, calculations indicate that at a ${\mathrm{CF}}_{4}$ gas flow rate of 400 sccm, the Si etching rate is approximately 4.1 nm/s, and the etch selectivity of Si relative to SiGe is about 44. When the ${\mathrm{CF}}_{4}$ gas flow rate is further increased to 500 sccm, the Si etching rate significantly rises to 6.0 nm/s, and although the etch selectivity of Si to SiGe slightly decreases to 41, it remains within an acceptable range. Therefore, it can be concluded that increasing the ${\mathrm{CF}}_{4}$ gas flow rate in the ${\mathrm{CF}}_{4}/O_{2}/N_{2}$ gas mixture not only achieves Sub-Fin tunability but also retains a high etch selectivity relative to SiGe. This experimental result strongly demonstrates that increasing the ${\mathrm{CF}}_{4}$ flow rate in the ${\mathrm{CF}}_{4}/O_{2}/N_{2}$ gas mixture effectively achieves controlled Si layer morphology while maintaining high etch selectivity. The mechanism is that the increase in ${\mathrm{CF}}_{4}$ flow rate directly leads to an increase in the relative number of active $F^{*}$ radicals in the etching environment, thereby strengthening the dominant role of the etching reaction and making the etching process more efficient and precise. This process alleviates the interference of stress effects on the etching results, ensuring the flatness and accuracy of the etched morphology [21,22].

## 4. Conclusions

This study presents an RPE-based Sub-Fin edit technology for the continuous tuning of the Sub-Fin profile in GAA FETs, enabling a transition from an arrow shape to a bell shape. This capability provides flexible optimization of device thermal management and performance across diverse application scenarios. The method achieves a maximum Si/SiGe selective etch ratio of 46, yielding well-defined post-etch SiGe layer edges with negligible degradation and precise control over the etch angle from 14° to 45°. The technology demonstrates excellent compatibility, tunability, and manufacturability, thereby offering a novel structural design and an engineering-viable pathway to effectively inhibit parasitic bottom transistor (trPBT) leakage, maintain superior heat dissipation, and ensure compatibility with advanced Si/SiGe epitaxial processes.

## Data Availability

The data presented in this study are available on request from the corresponding author.
